# The Association between Religiosity and Fertility Intentions Via Grandparenting: Evidence from GGS Data

**DOI:** 10.1007/s10680-023-09652-9

**Published:** 2023-02-15

**Authors:** Charalampos Dantis, Ester Lucia Rizzi, Thomas Baudin

**Affiliations:** 1 Center for Demographic Research, Institute for the Analysis of Change in Contemporary and Historical Societies, Université Catholique de Louvain, Ottignies-Louvain-la-Neuve, Belgium; 2 IESEG School of Management, Univ. Lille, CNRS, UMR 9221 - LEM - Lille Economie Management, F-59000 Lille, France

**Keywords:** Religiosity, Grandparental childcare, Fertility intentions, European countries

## Abstract

Although the literature concerning the association between religiosity and fertility in European countries is already quite extensive, studies exploring the mechanisms of action of religiosity are rare. The main aim of this article is to investigate whether grandparental childcare is a mediating or moderating variable in the association between attendance at religious services and the intention to have a second or third child. Building on previous literature, we assume that parents who are more religious might put more effort into establishing a positive relation with the grandparents of their child/children. Consequently, compared to parents who are less religious, those who are more religious could be more receptive to possible encouragement from grandparents to have another child and may be more optimistic regarding grandparents’ involvement with an additional child. Using Generations and Gender Survey (GGS) data for eleven European countries, we find evidence of a strong and positive effect of attendance at religious services on fertility intentions. Receipt of regular or weekly help from grandparents positively moderates the association between attendance at religious services and fertility intentions, albeit only for male respondents and mainly for the intention to have a second child.

## Introduction

Europe has been identified as “the continent with the lowest fertility” (The ESHRE Capri Workshop Group, 2010). The decline in fertility to a level below replacement across generations (2.1 children per woman) has been associated with the process of the second demographic transition (SDT) (Kertzer et al., 2009; Lesthaeghe, 2020 & 2010; Zaidi & Morgan, 2017). In addition to low fertility, the other main features of the SDT are increasing age at motherhood, an increase in divorce rates, and the proliferation of out-of-wedlock unions and births. This process began during the early 1960s and coincides with a profound change in the values of Western societies towards values of "self-realization" (Lesthaeghe, 2010). In this process, secularization—a decline in the influence of religion on social life and at the individual level—plays a major role (Lesthaeghe, 2010; Philipov & Berghammer, 2007; Voas, 2009).

Despite this considerable decline in religiosity, according to the Pew Research Center, in 2010, 81.2% of citizens of the European Union were affiliated with a religion, and 74.5% declared themselves to be Christians. Researchers have detected an increase in "believing without belonging" (Philipov & Berghammer, 2007: 276). The sociologist Grace Davie (1990) was the first to use the expression “believing without belonging” to describe the tendency of religious affiliation to remain at approximately the same level (or at a level that is only slightly reduced) during the second half of the twentieth century in Great Britain, while attendance at religious ceremonies declined rapidly.

At the macro level, the effect of religiosity on fertility remains important (Pew Research Center, 2015). However, the association between religiosity and fertility is complex, as many EU countries with high religiosity have very low birth rates (e.g. Poland, Italy, Greece and Romania). For this reason, researchers have focused mostly on the effects of religiosity at the micro level. Previous literature shows that at the individual level, religious practice exerts a strong and positive effect on fertility (Buber‐Ennser & Berghammer, 2021; Bein et al., 2020; Peri-Rotem, 2020; Bein et al., 2017; Peri-Rotem, 2016; Baudin, 2015; Régnier-Loilier & Prioux, 2015; Berghammer, 2012 and 2009; Régnier-Loilier & Prioux, 2008; Adsera, 2006).

Religious practice is also positively linked to strong family support (Dykstra & Fokkema, 2011), which is defined as “familialism”. In the case of intergenerational relations, Dykstra and Fokkema (2011:557) identify the following features of “descending” familialism: “living nearby, frequent contact, endorsement of family obligation norms, and primarily help in kind from parents to children”. Previous studies report a positive association between religiosity and grandparental childcare, especially when religiosity is measured via attendance at religious services (Dykstra & Fokkema, 2011; King & Elder, 1999). A positive relation between adult children’s religiosity and grandparenting is also plausible since the extant literature also shows that religion can strengthen intergenerational ties (Gans et al., 2009; Hwang et al., 2021; Pearce & Axinn, 1998). Moreover, the literature highlights the fact that grandparental childcare is positively linked with fertility (Taskanen et al., 2014; Taskanen & Rotkirch, 2014; Thomese & Liefbroer, 2013; Aassve et al., 2012; Waynforth, 2011; Kaptijn et al., 2010).

Based on these findings, the main aim of this article is to investigate whether grandparenting is a mediating variable in the association between religiosity and fertility. In other words, we posit that adults' children religiosity may be associated with grandparental help, which in turn is associated with fertility. Grandparenting could also be a moderating variable, indicating that the strength of the association between religiosity and fertility could vary depending on whether grandparents help with grandchildren (or not). In particular, we posit that parents who are more religious might put more effort into the task of establishing a positive relation with their child/children’s grandparents than would less religious parents.

Although the literature pertaining to the association between religiosity and fertility-related behaviour in European countries is already quite extensive (Bein et al., 2020; Bein et al., 2017; Peri-Rotem, 2016; Baudin, 2015; Berghammer, 2012 and 2009; Régnier-Loilier & Prioux, 2008; Adsera, 2006), studies exploring the mechanisms of action of religiosity, particularly the intervening role played by grandparental childcare, are rare.

Regarding our choice to study fertility intentions instead of actual fertility, few studies address the effect of religious practice on fertility intentions (Buber‐Ennser & Berghammer, 2021; Bein et al., 2017, 2020; Philipov & Berghammer, 2007). Compared to the actual number of children, fertility intentions are shaped more strongly by religious teachings (Charton et al., 2009; Hayford & Morghan, 2008), as the actual number of children might be more the result of economic constraints than cultural factors (Balbo & Mills, 2011; Hayford & Morghan, 2008).

Concerning parity of birth, we study the link between religiosity and the intention to have a second or third child. We do not consider the intention to have a first child. Grandparents’ availability and care ability with respect to the first and second child can be informative for parents and can affect their intentions to have another child; of course, such knowledge is not available prior to having the first child. Another reason to focus on the intention to have a second or third child is the fact that the effect of religiosity on the intention to have a first child may be underestimated due to stronger norms against nonmarital fertility and lower rates of nonmarital fertility among the most religious individuals compared to less religious individuals (Hayford & Morgan, 2008). An additional reason to focus on the intention to have a second or third child is the fact that the current low fertility rates in European countries are due mainly to the low percentages of couples who have second- or higher-order children (Philipov & Berghammer, 2007).

To measure the association between religiosity and fertility intentions, we use the first wave of the Generations and Gender Survey (GGS) for eleven European countries (Bulgaria, Russia, Georgia, Germany, France, Romania, Austria, Lithuania, Poland, the Czech Republic and Sweden) for which information concerning religiosity, religious affiliation, and grandparenting is available.

In this study, we focus on the association between men’s and women’s attendance at religious services and their intentions to have a second or third child. We also consider the mediating or moderator role played by grandparenting in the relation of interest. The structure of the article is as follows. First, in Sect. 2, we present the theoretical and empirical background alongside our hypotheses. In Sect. 3, we describe the data and methods as well as the operationalization of concepts. In Sect. 4, we present our findings. Finally, in the conclusion, we discuss our results and the limitations of our study.

## Theoretical Background and Previous Findings

### Religiosity and Fertility

According to McQuillan, religion proposes behavioural norms influencing fertility outcomes. To affect fertility, religious rules must be directly related to fertility, for example, by referring to contraception or abortion, but they can also be more general rules concerning gender roles and the organization of family life (McQuillan, 2004). Ceremonies that mark important life events, such as birth or marriage, might also represent an opportunity for Church representatives to identify conformity with religious teaching: denial of access to these rites compromises participation in a religious community (McQuillan, 2004). At the same time, when people choose to celebrate important life events with religious rites, they expose them-selves to religious teaching and norms.

Similarly, other studies emphasize the role of religious teachings on norms, the organization of family life, and, either directly or indirectly, fertility ideals and behaviours (Adsera, 2006; Baudin, 2015; Stonawski et al., 2015). Moreover, related studies stress the fact that religious teachings are conveyed in a context of socialization, where couples receive support from other members of their religious community (Myers, 2004; Philipov & Berghammer, 2007), and this situation is likely to increase the probability of these couples having high fertility ideals and intentions (Philipov & Berghammer, 2007).

In the extant literature, two main indicators are employed to capture religiosity: religious affiliation and attendance at religious services. Previous studies indicate a positive association between religious affiliation and fertility, particularly in the case of a third child (Baudin, 2015; Berghammer, 2009; Pew Research Center, 2015; Philipov & Berghammer, 2007). However, according to Adsera (2006), members of a religious denomination who do not actively participate are less likely to be “committed” to the denomination’s teachings, and attendance at religious services affects fertility more strongly than religious affiliation (see also Baudin, 2015; Berghammer, 2009; Philipov & Berghammer, 2007; McQuillan, 2004). Moreover, attendance to religious services increases the likelihood of contact with and support from the religious community mentioned above. Thus, religious attendance is the indicator of religiosity used in this study.

Some studies also show that men’s religiosity has a stronger effect on fertility ideals than women’s religiosity (Adsera, 2006; Hubert, 2015). This finding may be linked to the fact that the percentage of nonreligious individuals is higher among men than among women in most countries. The minority status of religious men probably indicates more conscious participation in religious services than typically exhibited by religious women. Consequently, it is no surprise that religious men exhibit more distinctive fertility behaviour than nonreligious men.

Thus, following previous studies, *we posit that religious practice is positively associated with fertility intentions (H1) and that this association is stronger for men than for women (H2).*

### Religiosity and Grandparenting

Religion can strengthen relationships among family members. At least three mechanisms have been identified (Pearce & Axinn, 1998). First, religious teaching emphasizes social solidarity, promotes intergenerational solidarity, and features rituals and activities that reinforce intergenerational ties (Gans et al., 2009; Pearce & Axinn, 1998). Second, religious institutions organize activities that promote interaction among family members. These activities include religious services where families spend time together as well as other family activities, such as family camps or retreats (Pearce & Axinn, 1998). Third, religious institutions, through activities and services, connect friends and family members in the same social group. It is reported that closure in social ties promotes strong parent–child relations (Coleman, 1988, cited by Pearce & Axinn, 1998).

Consistent with this theoretical framework, extant empirical studies indicate a positive association between religiosity and the quality of intergenerational relations (Gans et al., 2009; Hwang et al., 2021; Pearce & Axinn, 1998). These studies refer to the religiosity of parents or, alternatively, the religiosity of adult children to ascertain their effect on the quality of the relationship, on norms of solidarity, and on supportive behaviour across generations. Pearce and Axinn (1998) show that mothers' religiosity, measured at different moments throughout the child’s life course, has a positive effect on the quality of the relationship when the child is in early adulthood. Analogous results are obtained by King (2003) regarding fathers’ religiosity. Gans et al. (2009) investigate the effects of religiosity on filial norms and actual supportive behaviour towards parents, showing that religious adult children are more committed to and involved in parental care than less religious adult children. Similarly, Hwang et al. (2021), comparing Baby Boomers and Gen-Xers, find that in both generations more religious individuals express stronger filial norms than less religious individuals.

Interestingly, a positive association between the religiosity of grandparents and grandparental childcare is also detected (Dykstra & Fokkema, 2011; King & Elder, 1999), especially when religiosity is measured in terms of attendance at religious services (Dykstra & Fokkema, 2011). These studies consider the religiosity of grandparents, while in our study, only information on the religiosity of adult children is available. Is a positive association between adult children’s religiosity and grandparenting also plausible? Some arguments exist to support this association. A first such argument is based on the correlation between the religiosity of adult children and that of their parents (e.g. Bengtson et al., 2009; Glass et al., 1986; Myers, 1996; Petts, 2015). Due to this correlation, we could say that accounting for adult children’s religiosity is equivalent to considering grandparents’ religiosity. One objection to this equivalence could be that the transmission of religiosity weakens across generations. For example, it has been shown that in Austria in 2008, 71% of religiously unaffiliated persons had parents who were both religiously affiliated (Potančoková & Berghammer, 2014). Religiosity can also change over an individual’s life course: many religious persons have a sense of belonging in childhood and at the age of retirement but not during adulthood (Potančoková & Berghammer, 2014). Other factors, such as belonging to a stepfamily or a never-married single-parent family (Petts, 2015) or even the deterioration of the parental relation or the parent‒child relation (Leonard et al., 2013; Myers, 1996), might also mitigate the intergenerational transmission of religiosity. As a result, taking religious adult children as a proxy for grandparents’ religiosity might lead to bias due to the implicit selection of very religious parents who are more capable and motivated with respect to transmitting religiosity.

However, a second argument could be used to justify a positive association between adult children’s religiosity and grandparenting. Drawing on the literature presented above, adult children who are more religious could value more intergenerational ties and put more effort into the development of good relation quality with the older generation and into the encouragement of grandparent–grandchildren relations, thus promoting grandparental help. Moreover, due to the positive effect of religiosity on intergenerational support mentioned previously, religious adult children are more likely to be embedded in a dense web of mutually supportive behaviours with the older generation, and they are thus more likely to receive grandparental help with childcare.

### Grandparenting and Fertility

Help from grandparents can vary in terms of its intensity, but it has been shown to be a common practice in all national contexts (Hank & Buber, 2009). Previous studies show that grandparental support is positively associated with the fertility of adult children (Taskanen et al., 2014; Taskanen & Rotkirch, 2014; Thomese & Liefbroer, 2013; Aassve et al., 2012; Waynforth, 2011; Kaptijn et al., 2010). One possible explanation for this fact is that grandparents help parents balance work and family and thus allow them to consider the arrival of another child more positively.

Considering previous studies on the associations between religiosity and fertility, on the one hand, and those between religiosity and grandparenting, on the other hand, *we hypothesize that grandparental support in childcare is a mediating variable in the association between religious practice and fertility intentions, i.e. grandparenting partially mediates the effect of religiosity on fertility intentions (H3). Alternatively, we propose the existence of a moderation effect, i.e. that the effect of grandparenting on fertility intentions is greater among the most religious parents (H4).* As mentioned previously, due to their stronger familialism, religious adult children might put more effort into the tasks of establishing a positive family relation with their own parents and promoting the grandparent–grandchild relationship. As a result, they could be more receptive to encouragement from the older generation to have another child, and they may be more optimistic regarding grandparents’ involvement with an additional child.

## Data and Method

For the purposes of this paper, we use data from the GGS, which collects comparable and multidimensional microlevel data concerning fertility, partnership, generations, economic activity, and care duties. The first wave of the GGS was conducted between 2004 and 2013 in 20 countries. An average of 9,000 individuals per country were interviewed. The first wave is the only wave to include information concerning religiosity for a large number of countries. In particular, for this study, we use data from eleven European countries: Bulgaria, Russia, Georgia, Germany, France, Romania, Austria, Lithuania, Poland, the Czech Republic and Sweden. The selected countries are the only ones for which information concerning religious affiliation, attendance at religious services, and grandparenting is available. Other countries included in the GGS lack information pertaining to religion or religiosity; e.g. for Australia, Italy and Estonia, information related to religious affiliation is unavailable, while for the Netherlands and Hungary, data concerning attendance at religious services are lacking. In Italy, information pertaining to grandparenting is also unavailable.

We focus on men and women who have one or two children, who are aged 20–49 years and who are partnered (married or in cohabitation). Due to the small sample size at the country level, we pool the data from the eleven countries. After individuals with missing information are removed, the final sample consists of 7,043 individuals with one child and 9,104 individuals with two children. As in other surveys, this sample includes more female respondents than male respondents (Fokkema et al., 2016) either due to men's refusal to answer or due to their unavailability.

We investigate the relationship between religiosity and fertility intentions. For the dependent variable, we group the four categories—1 “definitely not”, 2 “probably not”, 3 “probably yes” and 4 “definitely yes”—of the variable “Intention to have a/another child during the next three years” into a binary variable, where a value of 1 represents a positive answer and a value of 0 represents a negative answer. Our reason for focusing on fertility intentions “during the next three years” is that individuals who declare their intention to have a child “within 3 years” have nearly decided to have another child. This answer reflects a real interest in having another child and reduces the possibility of a change in intentions (Hayford & Morgan, 2008).

Regarding religiosity, our main explanatory variable, by combining the variables related to attendance at religious services and religious denomination, we create a new variable containing three categories: (1) at least monthly attendance; (2) less than monthly attendance; and (3) no religious affiliation. In this study, we refer to this variable as both “attendance at religious services” and “religious practice”. We must highlight the fact that among the affiliated, Christian denominations represent 94.7% of the sample of parents with one child and 92.7% of the sample of parents with two children. Thus, our results mostly pertain to members of Christian denominations.

Concerning grandparental childcare, two options are available: either the variable pertaining to grandparents’ regular help or the variable focused on grandparents’ weekly help can be used. We use the variable concerning regular help from grandparents, as it represents the most common practice among parents: in our sample, 37.7% of parents with one child receive regular grandparental help, while only 19.5% receive weekly help. The GGS questionnaire asks the following: “Do you (also) receive regular help with childcare from relatives, friends or other people for whom caring for children is not a job?” In case of an affirmative answer, the survey also asks the following: “From whom do you receive this help?” This approach allows us to create a dichotomous variable (yes/no) concerning grandparents’ regular help. In the category of childcare by grandparents, we include childcare by great-grandparents, who help more than 8% of the couples included in our sample.

The control variables are respondents’ age, marital status, educational level, gender, activity status, country of residence, and age of youngest child. Respondents’ ages are recoded into four categories: (1) 20 to 29 years; (2) 30 to 34 years; (3) 35 to 39 years; and (4) 40 to 49 years. Concerning the age of the youngest child, we create a new variable containing three categories: (1) 0 to 1 year; (2) 2 to 4 years; and (3) 5 + years. With respect to the marital status variable, we combine the GGS variables *marital status respondent* and *current partner status*. This variable includes two categories: (1) married and (2) in cohabitation. Regarding educational level, we create a variable featuring three categories: (1) low (preprimary level, primary level, lower secondary level); (2) medium (upper secondary level, postsecondary nontertiary level); and (3) high (first stage of tertiary, second stage of tertiary). We recode the variable *activity status respondent* and create a new variable featuring three categories: (1) employed/self-employed; (2) unemployed; and (3) inactive. Another control variable is country of residence (see the list of countries presented above). Finally, the age of the youngest child is recoded into three categories: “0–1”, “2–4” and “5 + ”.

Regarding the estimation method, a logistic regression analysis is conducted to measure the association between the intention to have another child within three years and religiosity at the time of the survey (Kleinbaum et al., 2002). To find support for our hypotheses, we estimate models for each grouping: the sample of parents with one child and the sample of parents with two children (Table 2 in Appendix). To ascertain the mediating effect of grandparenting, we estimate models both excluding and including grandparental childcare: if the effect of religiosity on fertility intentions varies after grandparental childcare is added to the model, this result would represent initial evidence concerning the mediating effect of grandparenting.

A further model includes the interaction Religiosity*Grandparental childcare to investigate whether the effect of religiosity on fertility intentions is moderated by the receipt of regular help from grandparents (Table 3 in Appendix). Another model includes the interaction Religiosity*Gender to determine whether the effect of religiosity on fertility intentions varies with the gender of the respondent (Table 3 in Appendix).

**Fig. 1:**
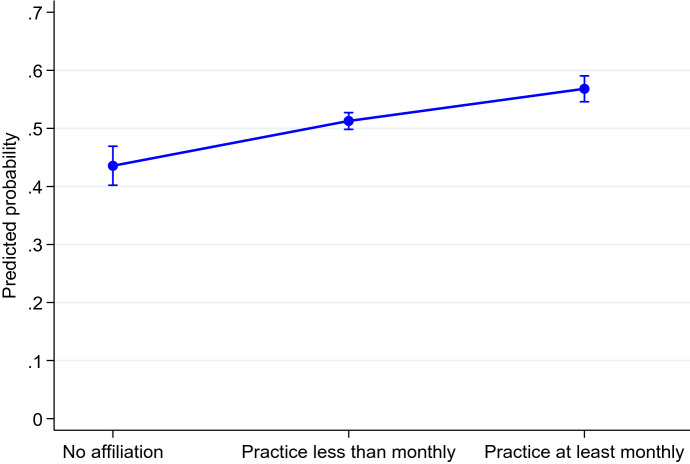
Predicted probability of intending to have a second child by religious practice. Note: Control variables are religiosity, age, marital status, educational level, gender, activity status, country, and age of youngest child; 95% confidence interval

**Fig. 2:**
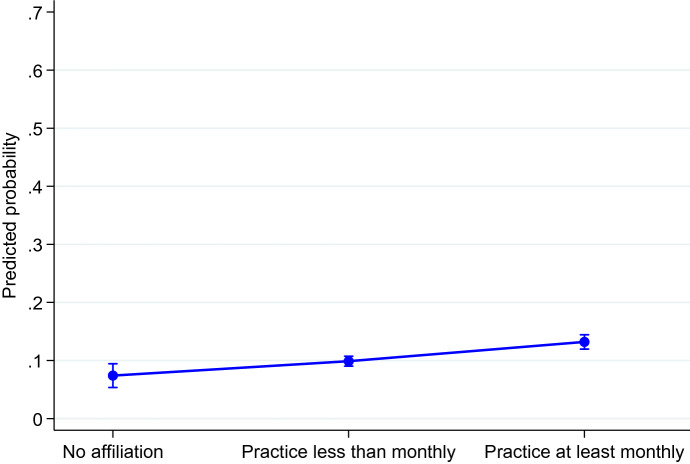
Predicted probability of intending to have a third child by religious practice. Note: Control variables are religiosity, age, marital status, educational level, gender, activity status, country, and age of youngest child; 95% confidence interval

**Fig. 3:**
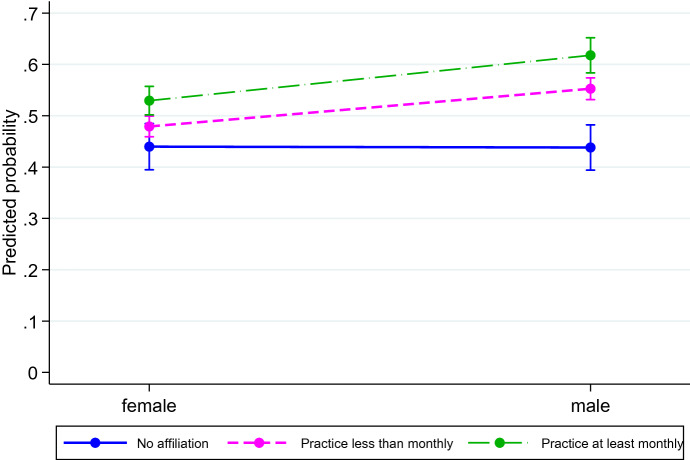
Predicted probability of intending to have a second child by religious practice and by gender. Note: Control variables are religiosity, age, marital status, educational level, gender, activity status, country, age of youngest child, and grandparental childcare; 95% confidence interval

**Fig. 4:**
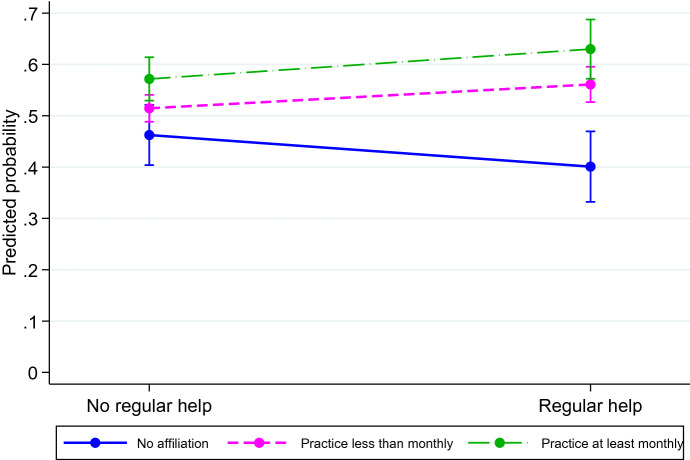
Predicted probability of intending to have a second child by religious practice and regular grandparenting (men only). Note: Control variables are religiosity, age, marital status, educational level, gender, activity status, country, age of youngest child, and grandparental childcare; 95% confidence interval

On the basis of these models, we estimate the odds ratios (OR) and the predicted probabilities of intention to have a second or third child according to religiosity (Figs. 1 and 2). These probabilities are also calculated according to the gender of the respondents (Fig. 3) as well as the availability (or nonavailability) of regular help from grandparents (Fig. 4).

To check the robustness of our results, the interaction models are estimated with respect to alternative samples (children younger than 10 years old and various national contexts). We also consider measures of the four categories of fertility intentions. Regarding the main explanatory variable, we try to use an alternative indicator of religiosity that is associated with the importance placed on religious ceremonies with respect to births, weddings or funerals (Table 4 in Appendix). Finally, to check the robustness of the results, in Table 5 (in Appendix), we employ the variable “weekly help from grandparents” instead of “regular help”.

## Results

### Descriptive Analysis

In the sample of individuals with one child, 51.8% of people intend to have another child within the next three years (Table 1 in Appendix). Regarding religiosity, 28.2% of parents of one child attend religious ceremonies at least monthly, 58.3% attend less than monthly, and 13.5% have no religious affiliation (Table 1 in Appendix). Concerning regular grandparental help with childcare, 38.3% of parents of one child receive regular help (Table 1 in Appendix). Finally, our descriptive analysis shows that the percentages of grandparenting do not vary according to level of religiosity (results not shown).

In the sample of individuals with two children, only 10.6% intend to have a third child within the next three years (Table 1 in Appendix). Concerning religiosity, 31% of parents of two children attend religious ceremonies at least monthly, 56.5% attend less than monthly, and 12.5% have no religious affiliation (Table 1 in Appendix). Descriptive statistics reveal that 31.6% of parents of two children receive regular help (Table 1 in Appendix); thus, fewer parents in this category receive help than do parents with one child. Finally, for respondents with two children, the percentages of grandparenting also do not seem to vary according to the levels of religiosity (results not shown).

### The effect of Religious Practice on the Intention to have a Second Child

We perform a logistic regression on the sample of individuals with one child. Our findings show that religiosity is positively associated with the intention to have a second child. This association is statistically significant (p < 0.01), and it is particularly strong among people who attend religious services at least monthly in comparison to people with no religious affiliation (OR = exp (0.63) = 1.88, Table 2 Model 2a in Appendix). The odds of intending to have a second child is also higher for people who attend religious services less than monthly than for those who have no affiliation (OR = exp (0.36) = 1.44, *p* < 0.01, Table 2, Model 2a in Appendix). To make these results more tangible, we also calculate predicted probabilities (Williams, 2012). For the most religious group, the corresponding predicted probability of intending to have a second child is nearly 60%. For individuals who attend religious services less than monthly, the corresponding predicted probability of intending to have a second child is approximately 50%. For nonaffiliated individuals, the predicted probability of intending to have a second child is approximately 45% (Fig. 1).

The interaction Religiosity*Gender is positive and statistically significant, indicating that the association between religiosity and fertility intentions varies according to the gender of the respondent (Table 3, Model 2a in Appendix). The corresponding probability of intention to have a second child for men who attend religious services at least monthly is more than 60%, approximately 55% for those who practise less than monthly, and 45% for nonaffiliated men (Fig. 3). Among women, the probabilities are lower, and differences across religious groups are not statistically significant (Fig. 3).

Adding the variable grandparenting does not change the results regarding religiosity, contradicting our hypothesis concerning the mediating role played by grandparenting (Table 2, Models 1a and 2a in Appendix). However, the interaction Religiosity*Grandparental childcare is positive and statistically significant (Table 3, Model 1a in Appendix). This result signifies that the association between religiosity and fertility intentions is moderated by regular grandparental help with childcare.

Interestingly, the interaction Religiosity*Grandparental childcare*Gender is also statistically significant, showing that the combined effect of religiosity and grandparenting on fertility intentions pertains only to men. In particular, Fig. 4 shows that men who engage in religious practices more than monthly and receive regular help from grandparents have a probability of intending to have another child of nearly 65%. This result is significantly different from the probability of intending to have another child among men who have no affiliation, which is equal to 40% (Fig. 4).

### The effect of Religious Practice on the Intention to have a Third Child

Religious practice is also positively associated with the intention to have a third child (Table 2, Model 2b in Appendix). As previously observed with respect to the intention to have a second child, people who attend religious services at least monthly have higher odds of having such an intention than those with no affiliation: OR = exp (0.67) = 1.95, *p* < 0.01 (Table 3 Model 2b in Appendix). For those who attend less than monthly, we obtain OR = exp (0.32) = 1.38, *p* < 0.05 (Table 3 Model 2b in Appendix). The corresponding probabilities are as follows: the probability of intending to have a third child is 13% for those who attend at least monthly, 10% for people who attend less than monthly, and 7% for individuals who have no religious affiliation (Fig. 2).

Similar to the findings concerning the intention to have a second child, for the intention to have a third child, adding the grandparental childcare variable to the model does not change the odds ratios for religiosity, thus contradicting our hypothesis regarding the mediating role played by grandparenting (Table 2 in Appendix). Finally, contrary to previous results on the intention to have a second child, the interaction terms are no longer significant, excluding a moderator role of grandparenting or gender in the link between religiosity and intentions to have a third child (Table 3 in Appendix).

### Robustness Check

Several robustness checks are performed. First, we select couples with young children, who are the couples who primarily benefit from grandparental care. Limiting our sample to parents with children up to 10 years old does not change our results (results available upon request).

Second, we estimate nested models showing that the positive effect of religiosity on fertility intentions increases slightly after the addition of control variables. In particular, adding country of residence changes the religiosity coefficient most substantially. A model featuring the interactions between religiosity and each country is also estimated. We expect to observe greater impacts from religiosity (representing encouragement from religious teachings and religious communities) and grandparental help on fertility intentions in countries in which family policies are limited; however, no consistent pattern is detected (results available upon request). We also run a model featuring interactions among religiosity, grandparenting, and country of residence. Again, no consistent results emerge (results available upon request).

Third, regarding our dependent variable, we use the original version of the variable featuring four categories for the intention to have a child during the next three years (“definitely not”, “probably not”, “probably yes” and “definitely yes”), and we estimate a multinomial logistic model using the same controls as in Table 2 (in Appendix). Overall, our previous results are confirmed. The risk that someone who practises at least once per month now answers “definitely intend” to the question concerning having a second child (rather than “definitely not intending to have one”) is 3.1 times the risk for someone who has no affiliation (in the case of multinomial logistic model results are expressed as relative risks). Regarding the intention to have a third child, the higher relative risk is observed for the category “probably yes”: the risk that someone who practises at least once per month “probably intends” to have a third child (rather than “definitely not intending to have one”) is 2.6 times the risk for someone who has no affiliation. Moreover, multinomial logistic model confirmed previous results of the binary logistic model concerning the interaction between religiosity and gender. The results of the binary logistic model interacting religiosity and grandparenting are also confirmed by the multinomial model when studying the intention to have a second child, while more contradictory results are obtained in the case of the intention not to have a third child (results available upon request).

Fourth, we try an alternative coding for the main explanatory variable: in addition to people with no religious affiliation, we include religious people who do not practise in the reference category. In this case, the interaction with regular grandparenting is no longer statistically significant. In other words, the joint effect of monthly attendance at religious services and grandparenting must be compared to the effect on individuals without a religious affiliation to detect a significant difference.

Fifth, we develop an alternative indicator of religiosity. Specifically, we consider three variables that measure the importance that respondents attach to religious ceremonies with respect to births, weddings, and funerals. Subsequently, we develop an indicator summing these three variables. Generally, our previous results hold true. The interaction between the importance attached to ceremonies and grandparenting is statistically significant for both the intention to have a second and a third child. The interaction between the importance attached to ceremonies and gender of the respondent is statistically for the intention to have a third child (Table 4 in Appendix).

Finally, a further model is estimated using a different indicator for grandparenting. In this supplementary analysis, we employ a dichotomous variable for grandparenting, according to which a value of 1 designates receipt of grandparental help at least weekly, while a value of 0 corresponds to the receipt of no help or less than weekly help. This criterion is more demanding than that used to construct the indicator in the previous analysis, according to which grandparenting is equal to 1 in the case of the receipt of regular help. With the new indicator for grandparenting, the results remain qualitatively similar (Table 5 in Appendix).

### Other Results

Individuals who cohabit without being married are more likely to intend to have a third child than are individuals who are married (Table 2, Models 2b in Appendix). This finding is in line with the conclusions of previous studies showing that many individuals who have two children and cohabit are divorced and desire a further child in a new marriage (Thomson et al., 2012). We conduct a descriptive analysis to confirm the higher propensity of divorced people who cohabit to intend to have a third child. In our study, among individuals who cohabit and have two children, 11% are divorced. Divorced persons who cohabit and have two children intend to have a third child in 29.8% of cases; the percentage of nondivorced cohabitants who intend to have a third child is 16.2%, while that of married persons who intend to have a third child is 9.6%.

We also observe that educational level is positively associated with intention to have a second or third child (Table 2 in Appendix). Some previous studies show no consistent results for parities higher than one; however, other studies show a positive educational gradient in Sweden (Berinde, 1999), Estonia (Klesment & Puur, 2010) and France (Köppen, 2006).

We observe that men are more likely to intend to have a second or a third child than are women (Table 2). In this respect, the extant literature is ambivalent, showing higher fertility intentions for men in some countries and higher intentions for women in other countries (e.g. Berrington, 2004; Lappegard et al., 2015).

Finally, inactive persons are more likely to intend to have a third child than employed and unemployed persons (Table 3, Model 2), echoing previous studies that show that women’s labour force inactivity is positively related to fertility (Cazzola et al., 2016; Cooke, 2009; Paihlé & Solaz, 2012).

## Discussion and Conclusion

This study explores the associations among fertility intentions, religiosity and grandparenting by consulting data from eleven European countries. Our *first hypothesis* proposed that attendance at religious services is positively associated with fertility intentions. This hypothesis was supported by our results: individuals who attend religious services at least once per month are more likely to intend to have a second or a third child than are those who have no religious affiliation. In other words, religious teachings promote pronatalist ideals, norms and behaviours (Adsera, 2006; Baudin, 2015; Stonawski et al., 2015), and individuals who attend religious services are more committed to these teachings and receive emotional and practical support from their fellow congregants (Myers, 2004; Philipov & Berghammer, 2007).

Our *second hypothesis* was also supported by our findings. Men who attend religious services at least monthly are more likely to intend to have a second child than are women, confirming previous results reported by Adsera (2006) and Hubert (2015). As highlighted above, this evidence may be linked to the fact that in most countries, religious men constitute a small minority (Adsera, 2006), which probably indicates more conscious participation in religious services than religious women as well as a more distinctive fertility decision process than that exhibited by men with no religious affiliation.

In our *third hypothesis*, we posited that grandparental support is a mediating variable in the association between religious practice and fertility intentions, and in our *fourth hypothesis,* we proposed that the effect of grandparenting on fertility intentions is greater when adult children attend religious services at least once per month. We found no evidence for the third hypothesis since the coefficients of religiosity do not change when accounting for grandparenting in the model. However, in line with our fourth hypothesis, we found evidence to suggest an interaction between religiosity and grandparenting, although this interaction only affected the intention to have a second child and pertained only to men. This is an original finding of our research as, to our knowledge, no previous studies have explored the mediator role of grandparenting.

How can the *combined effect* of religiosity and grandparenting on fertility intentions be explained? More religious adult children could value intergenerational ties more than nonreligious adults and could put more effort into the attainment of good relational quality with the older generation (Pearce & Axinn, 1998). For this reason, they might be more receptive to encouragement from their parents to have another child. Moreover, due to the good quality of these intergenerational relations, more religious adult children might be more confident in receiving grandparental help with a further child.

Why is this result observed *only in the case of men*? Previous literature shows that grandparents’ relational quality with their grandchildren is more strongly associated with the quality of their relationships with their children-in-law than with the quality of their relationships with their own children (Fingerman, 2004). Previous studies also show that daughters are more likely to receive help than are sons (Hank & Buber, 2009). Thus, the children-in-law who deal with grandparents are more frequently men. More than nonreligious men, religious men might desire a good-quality relationship with grandparents (Pearce & Axinn, 1998), and because of the efforts they make to achieve such a relationship, they could be more receptive to grandparents' encouragement to have another child and/or more confident that grandparents will care for the next child as well, thus resulting in the higher fertility intentions of these men with respect to having a second child.

Why is this result more consistently observed *with respect to the intention to have a second child*? Previous studies have shown that although grandparenting is beneficial to the health of grandparents (Danielsbacka et al., 2019), this benefit is not obtained when grandparenting is intensive (Arpino & Gómez-León, 2020). Moreover, the older age of grandmothers and grandfathers in the sample of parents with two children (whose average years of birth are 1946 and 1942, respectively) than that of grandmothers and grandfathers in the sample of parents with one child (whose average years of birth are 1949 and 1946, respectively) is a factor. Thus, encouragement to have a third child could be less likely to be given by these older grandparents.

One limitation of our study is the fact that only a limited number of countries are included in the sample. A larger number of countries would have allowed us to categorize the sample in accordance with the welfare state typology and verify the role played by the welfare state in the associations among religiosity, grandparenting and fertility intentions. Another limitation of the study pertains to the lack of information regarding the religiosity of grandparents. Parent–adult child religious discordance or concordance might affect fertility intentions more than adult children’s religiosity in isolation.

Another limitation of our study is the possibility of reverse causation: in the context of the effect of religiosity on fertility intentions, we cannot exclude the effect of fertility intentions on religiosity. Some studies based on panel data for the US have shown that the arrival of a child might affect parents’ religiosity (Argue et al., 1999; Ingersoll-Dayton et al., 2002; McCullough et al., 2005; Stolzenberg et al., 1995). The results pertaining to the EU are more controversial. Berghammer (2012) reports that having a first child does not predict a change in church attendance. The effect of fertility on religiosity has been explained in several ways. The arrival of a child could raise questions concerning the meaning of life, and parents could thus turn towards religion; parents might want to expose their children to positive religious values, thus exposing themselves to religion; the pro-family contents of religious teachings and rituals might attract large families; and the family-oriented services provided by religious groups (Berman et al., 2012) and the community support associated with religious service attendance (Lim & Putnam, 2010; Waite & Lehrer, 2003) can create additional incentives for households with a larger number of children to increase their levels of religious involvement (for a brief synthesis of the literature, see Dilmaghani, 2019). These explanations of the effect of the birth of a child on religiosity could also explain the effect of fertility intentions on religiosity. Parents who intend to have a child during the next three years might start to be interested in the meaning of life. They could also start worrying about education and the values that they desire to transmit to the unborn child. This spiritual search might expose them to religious teaching and thus increase their likelihood of attending religious services. Thus, reverse causation cannot be excluded. The cross-sectional structure of the GGS did not allow the examination of the direction of causality. Thus, the estimates reported in the previous sections reveal only the associations between the explanatory variables and the outcomes.

Despite these limitations, the originality of this study lies in the fact that it explores the mechanisms underlying the association between religiosity and fertility intentions while including grandparental help with childcare as a moderator. Inferring from our results and considering religiosity to constitute a proxy of the quality of the relationship between generations, we can say that the higher the quality of the relationship between grandparents and their son-in-law is, the stronger the effect of grandparenting on the son-in-law’s intentions to have a second child. Further studies including information on intergenerational relationship quality could provide support for our interpretation.

## Appendix

See Tables

**Table 1:** Descriptive statistics

		1-Child sample		2-Children sample	
		No	%	No	%
Intention of having a second child during the next 3 years	Does not intend	3,395	48.2	8,139	89.4
	Intends	3,648	51.8	965	10.6
Religiosity	At least monthly attendance	1,984	28.17	2,822	31
	Less than monthly attendance	4,105	58.28	5,143	56.49
	No religious affiliation	954	13.55	1,139	12.51
Age	20–29	2,307	32.76	1,014	11.14
	30–34	2,246	31.89	2,318	25.46
	35–39	1,591	22.59	3,101	34.06
	40–49	899	12.76	2,671	29.34
Marital status	Married	5,880	83.49	8,027	88.17
	Cohabitant	1,163	16.51	1,077	11.83
Educational level	Low	657	9.33	1,009	11.08
	Medium	4,156	59.01	5,579	61.28
	High	2,230	31.66	2,516	27.64
Gender	Male	3,203	45.48	3,928	43.15
	Female	3,840	54.52	5,176	56.85
Activity status	Employed/self-employed	5,676	80.59	7,110	78.1
	Unemployed	647	9.19	882	9.69
	Inactive	720	10.22	1,112	12.21
Countries	Bulgaria	1,144	16.24	1,307	14.36
	Russia	811	11.51	611	6.71
	Georgia	497	7.06	981	10.78
	Germany	429	6.09	612	6.72
	France	358	5.08	792	8.7
	Romania	969	13.76	855	9.39
	Austria	475	6.74	739	8.12
	Lithuania	625	8.87	668	7.34
	Poland	1,127	16	1,364	14.98
	Czech Republic	410	5.82	701	7.7
	Sweden	198	2.81	474	5.21
Age of youngest child	Less than 2 years	1,880	26.69	1,569	17.23
	2–5 years	1,988	28.23	2,225	24.44
	5 years or older	3,175	45.08	5,310	58.33
Grandparental childcare	Yes	4,345	61.69	6,225	68.38
	No	2,698	38.31	2,879	31.62
	**Total**	7,043	100	9,104	100

^1^,

**Table 2:** Coefficients of logistic regression models for the intention to have another child

	1-Child sample		2-Children sample	
	Model 1a	Model 2a	Model 1b	Model 2b
	Coeff	Coeff	Coeff	Coeff
Religiosity (Ref = No religious affiliation)				
At least monthly attendance	0.63***	0.63***	0.67***	0.67***
Less than monthly attendance	0.36***	0.36***	0.32**	0.32**
Age (Ref = 30–34)				
20–29	0.03	0.03	0.29***	0.29***
35–39	0.73***	0.72***	-0.53***	-0.53***
40–49	1.83***	1.83***	-1.18***	-1.18***
Marital status (Ref = Cohabitant)				
Married	0.18**	0.18**	-0.30**	-0.30**
Educational level (Ref = Low)				
Medium	0.00	0.01	-0.09	-0.09
High	0.28***	0.27***	0.35**	0.35**
Gender (Ref = Female)				
Male	0.32***	0.32***	0.45***	0.45***
Activity status (Ref = Inactive)				
Employed/self-employed	0.10	0.10	−0.20*	−0.21*
Unemployed	-0.12	-0.12	-0.04	−0.04
Countries (Ref = Bulgaria)				
Russia	−0.26***	−0.26***	0.84***	0.84***
Georgia	1.16***	1.18***	1.96***	1.96***
Germany	−0.02	−0.01	1.05***	1.05***
France	1.00***	1.01***	1.74***	1.74***
Romania	−1.17*	−1.15	1.18	1.18
Austria	0.54***	0.53***	1.44***	1.44***
Lithuania	0.16	0.18*	0.89***	0.89***
Poland	0.18*	0.18*	0.86***	0.86***
Czech Republic	0.68***	0.68***	1.19***	1.19***
Sweden	1.63***	1.63***	1.64***	1.64***
Age of youngest child (Ref = 0–1 year)				
2–4 years	-0.05	−0.05	-0.15	−0.15
5 + years	-0.71***	−0.70***	-0.59***	−0.59***
Grandparenting regular (Ref = No)				
Yes	0.08		0.01	
No. =	7,043	7,043	9,104	9,104
Log likelihood =	−4261.92	−4260.69	−2705.36	−2705.35

****p* < 0.01 ***p* < 0.05 **p* < 0.1

^2^,

**Table 3:** Coefficients of logistic regression models for the intention to have another child (with interactions)

	1-Child sample		2-Children sample	
	Model 1a	Model 2a	Model 1b	Model 2b
	coefficients	coefficients	coefficients	coefficients
*MAIN EFFECTS*				
Religiosity (Ref = No affiliation)				
At least monthly attendance	0.45***	0.51***	0.52***	0.54***
Less than monthly attendance	0.24**	0.18	0.28*	0.26
*INTERACTION TERMS*				
Religiosity*Grandparenting regular				
At least monthly attendance*Yes	0.30*		0.24	
Attendance less than monthly*Yes	0.23		0.02	
Religiosity*Gender				
At least monthly attendance*Male		0.45***		0.26
Attendance less than monthly*Male		0.36**		0.1
No. =	7,043	7,043	9,104	9,104
Log likelihood =	-4274.85	-4257.16	-2719.93	-2704.57

Note: Control variables are religiosity, age, marital status, educational level, gender, activity status, country, age of youngest child, and grandparental childcare. ****p* < 0.01 ***p* < 0.05 **p* < 0.1

^3^,

**Table 4:** Coefficients of logistic regression models for the intention to have another child (interactions using the importance of religious ceremonies variable)

	1-Child sample		2-Children sample	
	Model 1a	Model 2a	Model 1a	Model 2a
	coefficients	coefficients	coefficients	coefficients
*MAIN EFFECTS*				
Importance of religious ceremonies	0.05***	0.06***	0.04***	0.05***
*INTERACTION TERMS*				
Importance of religious ceremonies*Grandparenting regular				
Importance of religious ceremonies*Yes	0.04**		0.07***	
Importance of religious ceremonies*Gender				
Importance of religious ceremonies*Male		0.02		0.04*
No. =	7,002	7,002	9,057	9,057
Log likelihood =	-4230.10	−4231.64	−2688.49	−2691.09

Note: Control variables are religiosity, age, marital status, educational level, gender, activity status, country, age of youngest child, and grandparental childcare. ****p* < 0.01 ***p* < 0.05 **p* < 0.1

^4^,

**Table 5:** Coefficients of logistic regression models for the intention to have another child (interactions using the grandparenting weekly variable)

	1-Child sample	2-Children sample
	Model 1	Model 2
	coefficients	coefficients
*MAIN EFFECTS*		
Religiosity (Ref = No affiliation)		
At least monthly attendance	0.55***	0.65***
Less than monthly attendance	0.28***	0.35**
*INTERACTION TERMS*		
Religiosity*Grandparenting weekly		
At least monthly attendance*Per week	0.37*	0.12
Less than monthly attendance*Per week	0.42**	−0.23
No. =	7,043	9,104
Log likelihood =	−4257.53	−2703.00

Note: Control variables are religiosity, age, marital status, educational level, gender, activity status, country, and age of youngest child. ****p* < 0.01 ***p* < 0.05 **p* < 0.1

^5^
